# Long distance calls: Negligible information loss of little auk social vocalisations due to high frequency propagation losses

**DOI:** 10.1371/journal.pcbi.1011961

**Published:** 2024-12-02

**Authors:** Anna N. Osiecka, Przemysław Bryndza, Elodie F. Briefer, Katarzyna Wojczulanis-Jakubas

**Affiliations:** 1 Department of Vertebrate Ecology and Zoology, Faculty of Biology, University of Gdańsk, Gdańsk, Poland; 2 Behavioural Ecology Group, Section for Ecology and Evolution, Department of Biology, University of Copenhagen, Copenhagen, Denmark; 3 Institute of High Frequency Technology, Faculty of Electrical Engineering and Information Technology, RWTH Aachen University, Aachen, Germany; University of Toronto, CANADA

## Abstract

How well does the information contained in vocal signals travel through the environment? To assess the efficiency of information transfer in little auk (*Alle alle*, an Arctic seabird) calls over distance, we selected two of the social call types with the highest potential for individuality coding. Using available recordings of known individuals, we calculated the apparent source levels, with apparent maximum peak sound pressure level (ASPL) of 63 dB re 20 μPa at 1 m for both call types. Further, we created a sound attenuation model using meteorological data collected in the vicinity of the little auk colony in Hornsund, Spitsbergen. Using this model, we modelled the calls to reflect higher frequency filtering and sound level loss occurring during spherical spreading in perfect local conditions, down to the putative hearing threshold of the species, calculated to equal ASPL of signals “propagated” to roughly one kilometre. Those modelled calls were then used in a permuted discriminant function analysis, support vector machine models, and linear models of Beecher’s information statistic, to investigate whether transmission loss will affect the retention of individual information of the signal. Calls could be correctly classified to individuals above chance level independently of the distance, down to and over the putative physiological hearing threshold. Interestingly, the information capacity of the signal did not decrease with its filtering and attenuation. While this study touches on signal properties purely and cannot provide evidence of the actual use by the animals, it shows that little auk signals can theoretically travel long distances with negligible information loss, and supports the hypothesis that vocalisations could facilitate long-distance communication in the species.

## Author summary

The social calls of the little auk are individually distinctive. We looked at whether and how the information carried by these calls might change when their higher frequency components are filtered as the sound travels through the environment. To do so, we used recordings of known individuals in a spherical spreading model with atmospheric attenuation based on the local meteorological conditions, which weakened and filtered the signals. Interestingly, the information capacity of the signal did not decrease with its filtering and attenuation. While this study touches on signal properties purely, it shows that little auk signals can theoretically travel long distances with negligible information loss.

## Introduction

The ability to recognise one’s social partner–e.g. offspring, mate, or neighbour–is necessary to maintain stable social bonds. Colonial animals, such as seabirds, often rely on vocal cues to find each other in crowded aggregations [1–5]. But how reliable is the information content carried by acoustic signals at a distance?

While under some conditions, acoustic signals can travel over extreme distances (e.g. a blue whale’s (*Balaenoptera musculus*) song theoretically travelling through the oceans), this is not always the case. The propagation of a soundwave, i.e. how it moves through and changes in an environment, depends on a number of factors. First of all, signals of lower amplitudes will degrade much faster due to spherical spreading, than louder sounds. Additionally, as the sound propagates, its higher frequency content will be gradually filtered out, leaving only the lower frequency components at larger distances (and finally filtering these as well). How exactly this filtering will occur, and how fast a soundwave will travel, will be impacted by the medium in which it is traveling–its density, humidity, pressure, and more. At some point, a signal’s amplitude will be so low, and/or its frequency content so degraded, that it will no longer carry the information first encoded in it by the sender–and of course, as a result, the receiver will not be able to decode it.

**Fig 1:**
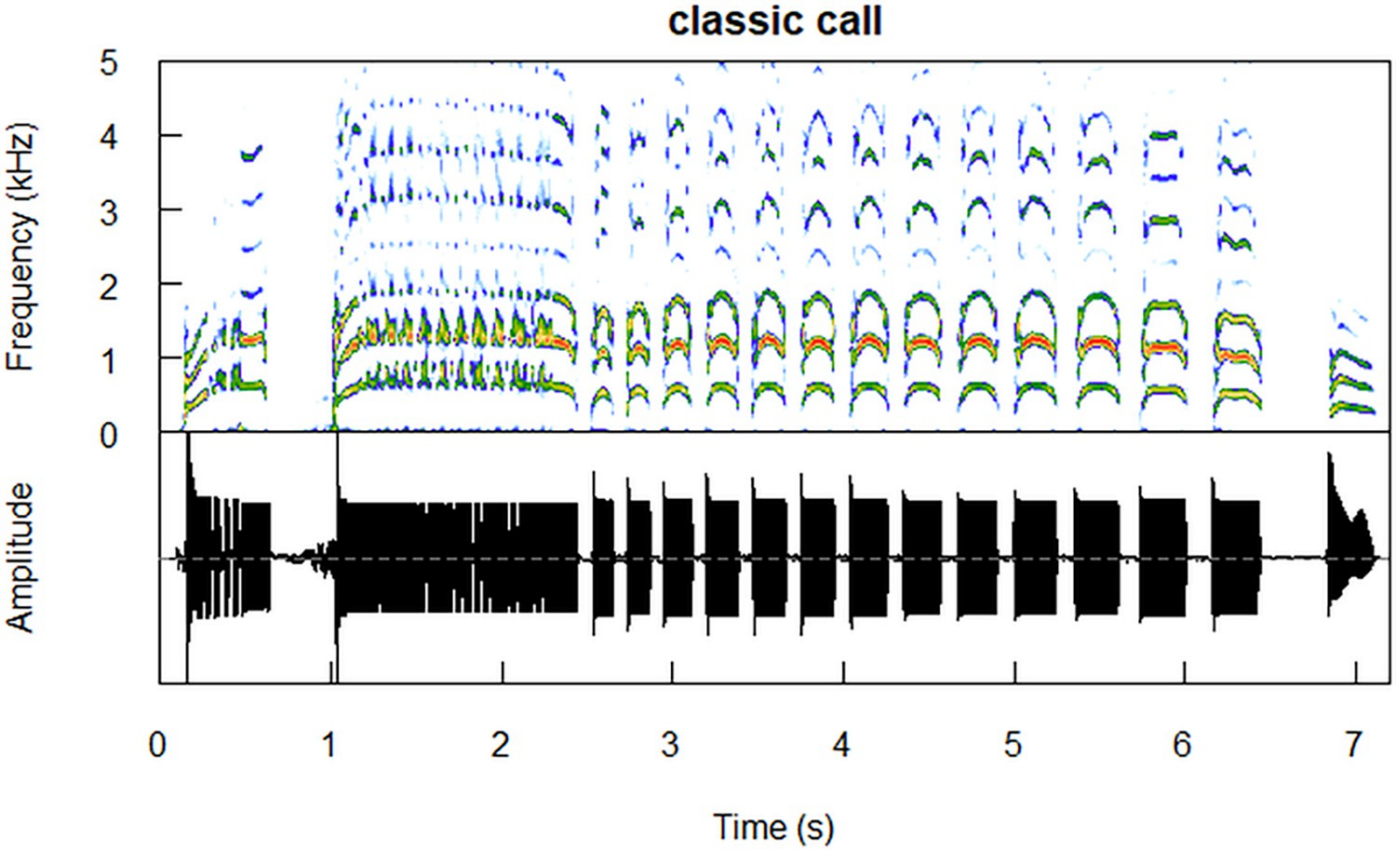
A sample *classic* call produced by an adult male (ring no. DA48567). Analysing bandwidth = 93.75 Hz. Spectrogram plotted using the *seewave* package [12].

Little auks (*Alle alle*) are highly colonial seabirds navigating complex social networks [6]. Little auks are also very vocally active [7], and their calls can carry a richness of static [8–10] and dynamic [7,11](information. The most complex call of the little auk repertoire, the *classic* call, is a long, compound signal with apparent formants, composed of a series of three types of syllables (Fig 1) [7]. It is a social call produced in a range of contexts, both by animals sitting inside their rocky nest chambers, and in flight, e.g. by birds returning to the colony from the foraging grounds [7]. While it carries no information on the caller’s sex or size, nesting partners tend to match certain properties of their *classic* calls [9]. This vocalisation carries reliable information on the sender’s identity, mostly within its spectral centre of gravity, fundamental frequency, duration, amplitude modulation rate, and frequency variation (in this order [10]), and has a higher information capacity than any other call type of the species [10]. The *classic* call likely plays a role in long-distance communication, possibly facilitating coordination of social behaviour. Therefore, it is likely to remain stable over behaviourally useful distances.

**Fig 2:**
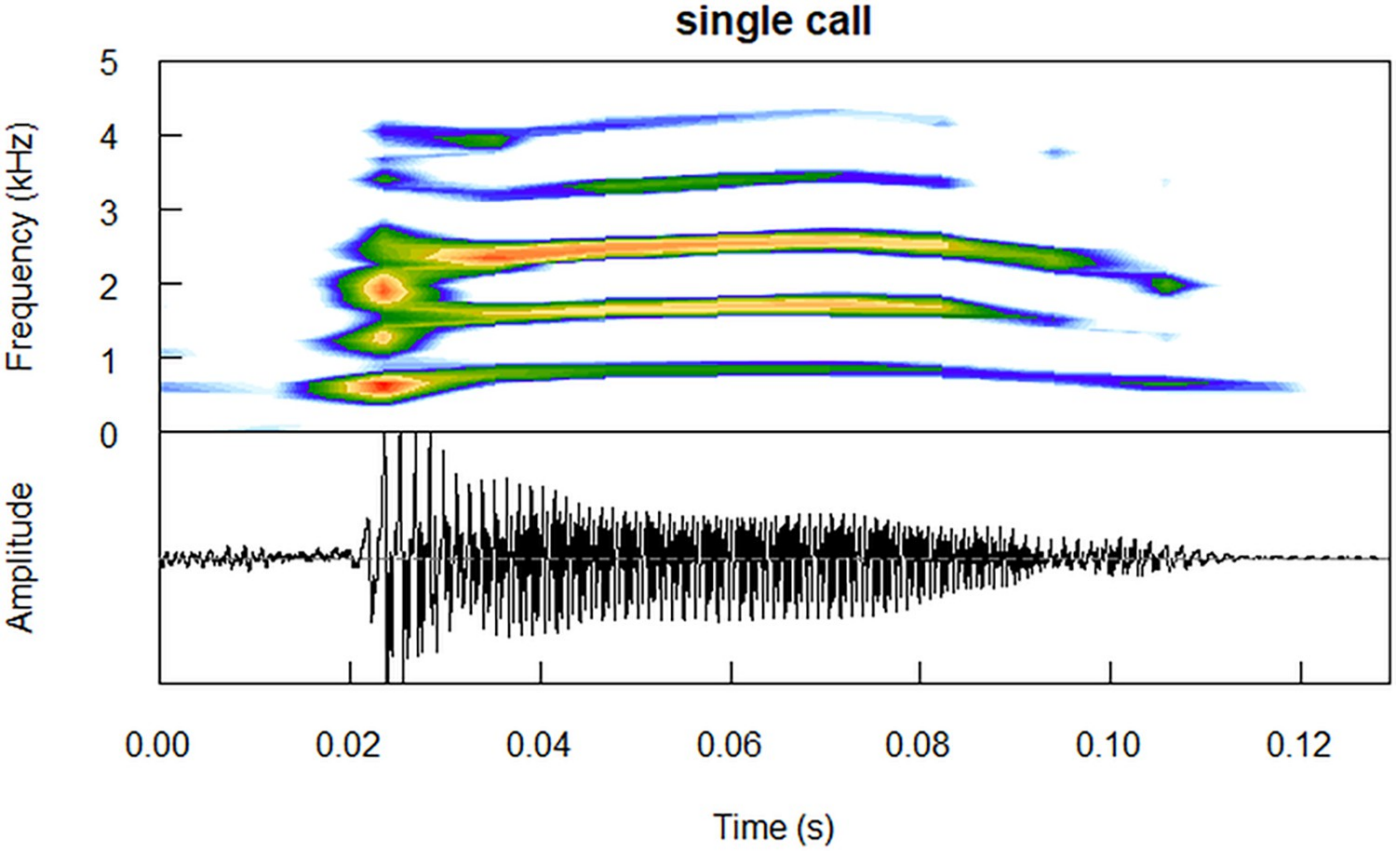
A sample *single* call produced by an adult male (ring no. DA48567). Analysing bandwidth = 93.75 Hz. Spectrogram plotted using the *seewave* package [12].

Another social call emitted in a range of situations, and both inside the nest and in flight, is the *single* call; a brief, one-syllable vocalisation (Fig 2) [7]. Like all little auk call types, this call is highly individually specific, and can be classified to an individual with the highest precision among all call types [10]. While the exact function of this call remains unknown, due to its short duration (less than 0.5 s) [7] and simple structure, it can be expected to serve in short-distance communication.

Here, we investigated how well the identity information encoded in the *classic* and *single* calls, which are both used by little auks throughout the entire breeding season, is maintained as the signal is attenuated in local atmospheric conditions, purely from a signal perspective, i.e. the transmission-related changes to carrying capacity of the vocal communication channel. We expected the *classic* calls to maintain the information content better than the *single* calls, which likely serve short-distance communication. To test this, we created a theoretical sound attenuation model using local meteorological data and a spherical spreading model, and, using sample calls of the two aforementioned types recorded from known individuals, we simulated call attenuation down to the putative physiological hearing threshold. We then investigated the information content of those modelled calls.

## Methods

### Ethics statement

This study used previously published data in theoretical models, and did not involve direct contact with the animals. Fieldwork involved in previous data collection was performed under permit from the Governor of Svalbard (20/00373-2), following the Association for the Study of Animal Behaviour’s guidelines for animal research.

All analyses were performed in Python v. 3.11 [13] and R environment (v. 4.1.3) [14], and full codes together with raw data have been provided in the supplementary materials (DOI 10.17605/OSF.IO/ESBDJ). Visualisations use scientific colour palettes [15–16] from package *khroma* [17].

### Study site and subjects

This study used previously published acoustic recordings (see detailed description below [10]). These recordings were collected during fieldwork in Hornsund, Spitsbergen, Norwegian High Arctic, over the incubation period in 2019–2020. This included handling (e.g. colour-ringing and measuring) the birds for standard ornithological procedures by a licensed ringer (KWJ, permit no. 1095, type: C, issued by Museum Stavanger, Norway), in order to be able to identify the focal individuals (see description below in the *Acoustic data* section). This study focused on 18 nesting pairs, i.e. 36 birds in total.

The study colony in Hornsund is comprised of the lower: 59–90 m a.s.l. and upper plot of the colony: 122–172 m a.s.l. Little auks maintain their flight height above their colony plots, and only descend for landing. For the purpose of this study, we selected 100 m as a representative flight height for the lower plot, i.e. the animals recorded in this study. This choice to select a flight height lower than the upper plot was made as a conservative measure to avoid accidentally increasing the modelled active space of little auk sounds (see model details below).

### Acoustic data

Audio material was collected via an Olympus ME-51S stereo microphone (-40 dB sensitivity at 1 kHz, frequency response 100–15,000 Hz +/-3 dB) placed inside each nest (a rock crevice/chamber, with floor covered with pebbles [6]) at approximately 10 cm from the birds inside, in such a way as to not disturb the birds’ normal activities. Each microphone was connected to an Olympus LS-P4 digital voice recorder (sampling rate 48 kHz, 16 bits, high gain) placed outside of the nest chamber and hidden under a rock to prevent both disturbance of the animals and damage to the equipment. Each nest was recorded three times over the incubation period, with recording sessions lasting 48 h and spaced about equally in time (i.e., around eight days in between recording sessions).

Sound recordings were paired with video monitoring of the nest entrance, so that we could see the birds entering and exiting their nesting chambers and extract the times at which only one known (ringed with a unique colour code) individual was present inside the nest chamber. Audio recordings from those periods were manually processed, resulting in the acoustic database of vocalisations produced by known individuals inside the nest. For more details on the field procedures, refer to Osiecka et al. 2024 [10].

### Apparent sound pressure level

To calculate the real-life sound pressure levels from the collected recordings, we first calibrated the equipment. First, a class II sound level meter (Volcraft SL-451) with no active filters applied was calibrated with a class II sound level calibrator (Volcraft SLC100) following instructions provided by the producer. Then, a 1 kHz tone was played using a JBL Flip 5 loudspeaker placed at 1 m from the recorder and sound level meter, and recorded with the same equipment and set-up as used in the field recordings. The obtained recording was used in end-to-end calibration of all digital audio recordings in Raven Pro 1.6.5, following the software specifications (https://ravensoundsoftware.com/knowledge-base/calibrating-recordings-in-raven-pro/).

Back-calculated sound pressure levels are termed *apparent sound pressure levels* (hereafter ‘ASPL’ [18]) to differentiate them from *sound pressure levels* (SPL) measured directly at 1m. ASPL (dB rms re. 20 μPa) at 10 cm of each vocalisation was extracted in Python using *numpy* package to obtain peak (i.e. the highest absolute magnitude of the signal) and root-mean-square (RMS, i.e. the RMS amplitude over signal duration, using the 95% energy threshold criterion [19]) values. The ASPL at 1 m, i.e. the Source Level (SL), was calculated as:

$$1.\;{SL}_{1m}={ASPL}_{10cm}-{20log}_{10}(\frac{100cm}{10cm})$$

To estimate a global mean of the ASPL values at 1 m, we first calculated the mean ASPL value for each individual, followed by a population mean. This was done for both call types, with peak and RMS values used separately. The obtained mean values were then compared between the call types using Welch two sample t-test (function *t*.*test*).

### Meteorological data

**Fig 3:**
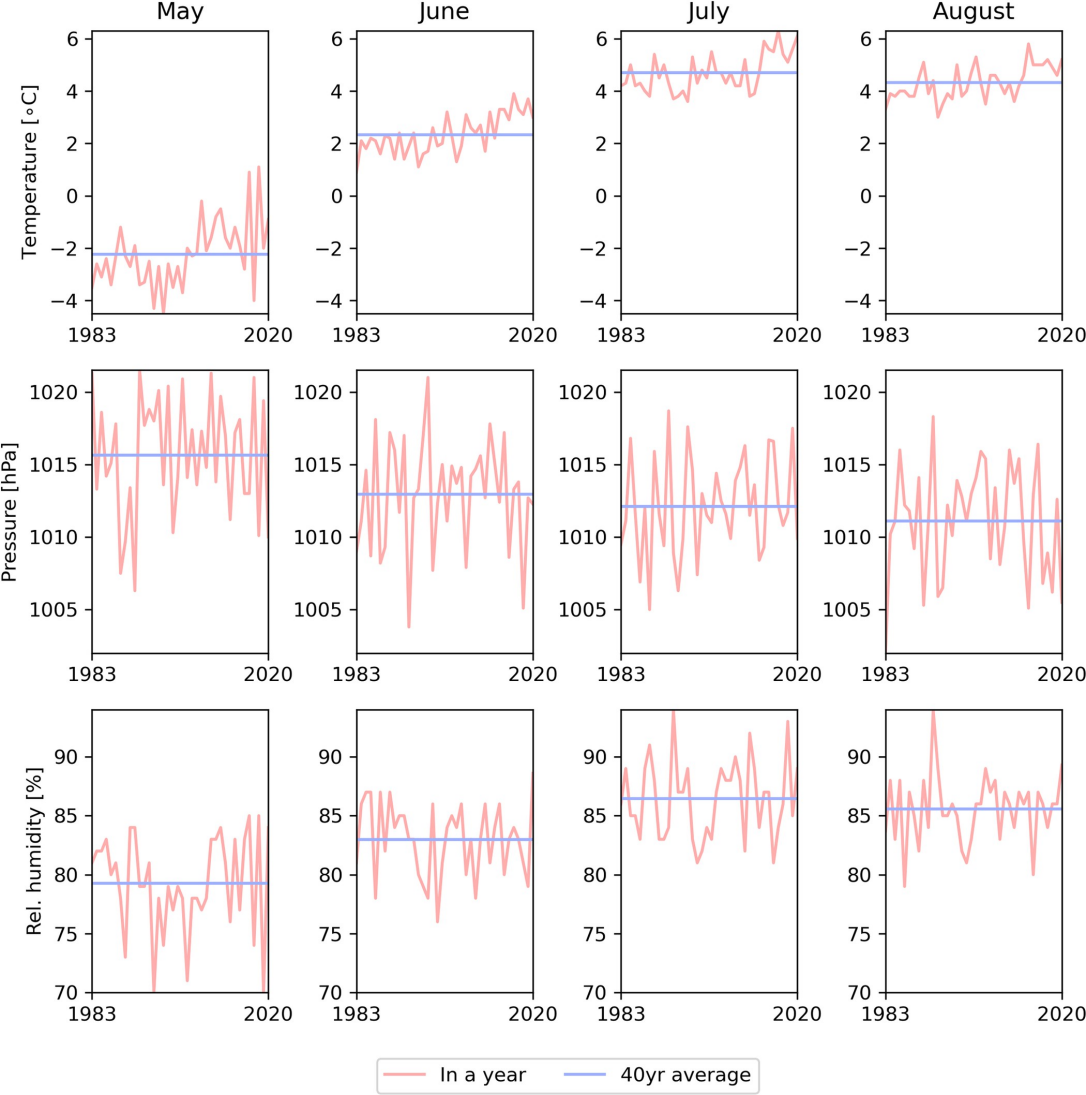
Annual (pink) and 40-year average (blue) meteorological data from Hornsund over the little auk’s breeding period (May-August).

Long-term geosystem monitoring data are publicly available from the Polish Polar Station in Hornsund, Institute of Geophysics, Polish Academy of Sciences (https://monitoring-hornsund.igf.edu.pl). For the purpose of this study, we selected data from 1983–2021, for which full meteorological information was available (as per August 2023, when the analysis was performed), focusing on May-August, i.e. the breeding period of the little auk [6]. Because those months are characterised by very different mean temperature, pressure, and relative humidity values (Fig 3) – and therefore different sound attenuation properties – we considered each month separately, using the 40-years average of each month in the following analyses.

### Attenuation model

To model attenuation of signals over distance, we used a spherical spreading model with the atmospheric absorption factor α based on the ISO 9613–1 standard [20]. The spherical spreading model describes how the energy of different frequency components of the signal changes over distance, working somewhat as a low-pass filter (i.e. the energy content of higher frequencies is lost earlier over propagation).

Note that this model comes with necessary simplifications: that is, it assumes simple spreading in idealised conditions, i.e. without added noise, in the absence of wind, and excluding excess attenuation. Simple spherical spreading was chosen based on the following: (1) We decided to model attenuation of calls produced in flight, and not in the nest, to simplify the model. Therefore, the signal source is an individual bird in flight, that is roughly 100 m over ground. This model is hence simplified to omit the impact of local topography on sound propagation (see [21]). While the *classic* and *single* calls are frequently produced both in flight and inside the nests, note that the calls used here were recorded inside the nest, since this is the only way we could control for the birds’ identity. The implications of this are addressed in the Discussion; (2) The Hornsund ornithogenic tundra is an open habitat with a dense vegetation cover composed of plant species reaching a maximum of approximately 20 cm in height [22], and is therefore expected to minimally degrade acoustic signals [23]; (3) The dense vegetation cover creates a soft substrate, so contribution of reflections is expected to be minimal; and (4) diel variations in meteorological conditions during the Arctic day are dictated by sea ice conditions rather than time day-night cycles [24], which means that reflections from different layers of the atmosphere are also expected to be minimal.

**Fig 4:**
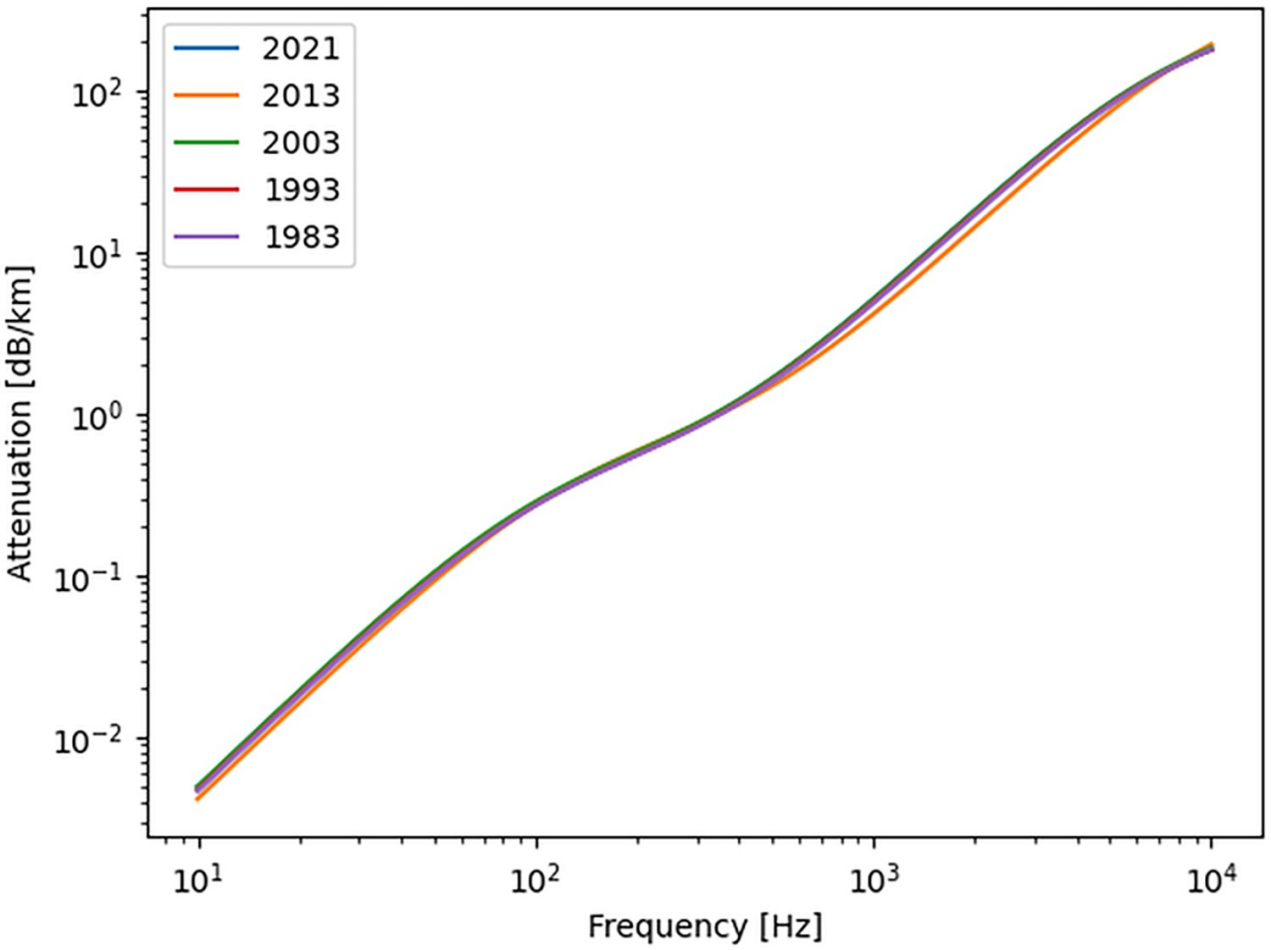
Sound attenuation at different frequencies, calculated from mean May conditions in Hornsund over the monitored period 1983–2021, based on the ISO 9613–1 standard [20]. There is no apparent shift in attenuation profiles over the years.

The ISO 9613–1 standard [20] gives fitted equations for atmospheric attenuation α as a function of frequency that is dependent on temperature, pressure and relative humidity of the air. The model is valid at altitudes below 10,000 meters, and so well within our case. As described above in the Meteorological data section, we used the local mean monthly values of relevant parameters, and subsequently α was calculated on those mean monthly values. We used the average values of the entire monitored period (1983–2021) rather than climate change-related patterns, since there was no apparent change in sound attenuation properties over the decades (Fig 4).

The resulting spherical spreading model is given by the following equation:

$$ASPL(r,f)={ASPL}_{1m}\frac{1m}{r^{2}}e^{-\alpha (f)r}[dB\;re\;20\;\mu Pa]$$

Where *e*^*x*^ is the natural exponential function, r is the distance (in metres), and α is a function of frequency as per ISO 9613–1 [20]. The full code of the attenuation model is available in the Supplementary Materials (DOI 10.17605/OSF.IO/ESBDJ; files atmospheric_attenuation.py and fiter_signal.py).

### Choice of the modelled distances

Since there is currently no information available on the hearing thresholds of the little auk, we used the in-air auditory measurements of another, related diving alcid species, the Atlantic puffin (*Fratercula arctica*), as a reference. The average physiological hearing threshold (measured using auditory evoked potential methods) in the alcids seems relatively similar across species, namely down to 10–20 dB re 20 μPa in the 1–2.5 kHz frequency range for the Atlantic puffin [25], down to 13 dB re 20 μPa in the 1–3.5 kHz range in the common murre (*Uria aalge* [26]), and down to 17 dB re 20 μPa in the 1–3.5 kHz range for the marbled murrelet (*Brachyramphus marmoratus [27]*). We chose 1000 m as the maximum modelled distance, with calculated ASPL at this distance roughly corresponding to the minimum physiological hearing threshold (i.e. the lowest SPL within the studied frequency range that still elicited brain activity during experimental procedures) of the Atlantic puffin [25]. Note that this does not translate to the active space of the signal, or how far away they could still be recognised by the auks (which would require a dedicated playback experiment to know), but rather as a guide to choose the modelled signal structure.

The attenuation model used calibrated recordings of known individuals at 10 cm as input files. Each file was modelled in meteorological conditions for May-August separately to mimic the signal structure corresponding to noise-free propagation to 1, 2, 4, 10, 21, 46, 100, 215, 464, and 1000 m (from here on, 1–1000 m), creating a separate audio file as an output. In other words, each original call was modelled at 10 distances in mean conditions of four separate months, that is 40 times in total. Note that this does not mean performing actual propagation experiment in the air, but purely mathematical modelling resulting in selectively filtered and attenuated vocalisations.

### Acoustic analysis

All obtained (i.e. modelled) audio files were batch-processed in R, using the *soundgen* package [28] (function *analyze* with settings adjusted to the little auk: dynamic range = 60 dB, pitch floor = 500 Hz, pitch ceiling = 2000 Hz, step = 5 ms) to extract a set of 15 acoustic parameters (Table 1). Both raw audio and the resulting analysed datasets can be found in the supplementary materials.

**Table 1:** Raw acoustic parameters extracted from audio files. Variable explanations as per *soundgen* package [28].

Variable	Definition
Duration	duration from the beginning of the first non-silent short-time Fourier transform (STFT) frame to the end of the last non-silent STFT frame [s]
AM Env Dep mean	depth (0 to 1) of amplitude modulation estimated from a smoothed amplitude envelope
AM Env Freq mean	frequency [Hz] of amplitude modulation estimated from a smoothed amplitude envelope
AM Ms Freq mean	frequency of amplitude modulation
Ampl mean	root mean square of amplitude per frame
CPP mean	Cepstral Peak Prominence [dB]
Dom mean	lowest dominant frequency band [Hz]
FM Dep mean	depth of frequency modulation
Peak Freq mean	the frequency with maximum spectral power [Hz]
Pitch mean	post-processed pitch contour based on all F0 estimates [Hz]
Q25%	the 25^th^ quantile of the spectrum of voiced frames [Hz]
Q50%	the 50^th^ quantile of the spectrum of voiced frames [Hz]
Q75%	the 75^th^ quantile of the spectrum of voiced frames [Hz]
Spec Centroid mean	the centre of gravity of the frame’s spectrum, first spectral moment [Hz]
Spec Slope mean	the slope of linear regression fit to the spectrum [dB/kHz]

The dataset was first cleaned, i.e., entries with missing values (that is, raw acoustic parameters that could not be correctly extracted) removed. We also reduced the dataset to the individuals with at least 200 entries (i.e., at least five calls propagated four times to 10 distances). This reduced the dataset to 5521 *classic* call entries from 11 individuals, and 2640 *single* call entries from six individuals.

To reduce data dimensions for further analyses, this cleaned dataset was subsequently tested for Kaiser-Meyer-Oklin factor adequacy (function *KMO*, package *EFAtools [29]*; S1 Table), and then used in a Principal Components Analysis (PCA; function *prcomp*, package *stats [30];* S2 and S3 Tables). This was done separately for each of the two call types.

### Classification to individual

To check how well the modelled calls can be classified to the caller independently of the higher frequencies filtering, we performed the following analysis. For each call type, we selected the principal components with eigenvalues > 1 (S2 Table) as input variables. The corresponding PC scores of all attenuated calls for which we were able to extract the full set of acoustic parameters specified in Table 1 were used in a permuted discriminant function analysis (pDFA [31]), to see how well can calls be classified to the caller independently of signal degradation. This pDFA was conducted in a nested design, using the *pDFA*.*nested* function (R. Mundry, based on function *lda* of the *MASS* package [32]), on all available calls (5521 for the *classic* call, and 2640 for the *single* call) of all the subjects (11 for the *classic* call, and six for the *single* call). Since the same calls were modelled in conditions corresponding to the four focal months (May-August), we used the file name as a control factor to correct for multiple sampling. We ran a total of 1000 permutations for the analysis. This was done separately for the two call types, for all modelled distances pooled together and each distance separately.

Furthermore, to see how well the attenuated calls cluster to individuals, we performed a set of additional analyses using support vector machine (SVM) classifiers. First, to establish the approximate number of nearest neighbours to use, we used the *kNNdistplot* function of the *dbscan* package [33]. We then reduced the data dimensions of the raw, cleaned datasets using supervised uniform manifold approximation and projection (S-UMAP; *uwot* package [34], *umap* function), with minimum distance = 0.5, n_neighbours = 500 (*classic*) or 200 (*single*), using the Euclidean metric. This gave us two-dimensional coordinates, subsequently introduced to the SVM classifiers. This approach was selected as the one that gave the best results in a previous study of vocal individuality in the species [10], and confirmed to yield the highest accuracy with the present dataset. The data were first subset into distances, and subsequently into 8:2 training:test datasets. A classification task was built for each subset (*mlr* package [35], function *makeClassifTask* with individual ring number as target). A learner was then created using *makeLearner* function of the *mlr* package [35], and corrected for individual weights due to the uneven sampling of different individuals (*mlr* package [35], *makeWeightedClassesWrapper* function). The weighted learner was then trained (*mlr* package [35], *train* function) on the training task, and used to classify the task (*mlr* package [35], *predict* function). Classification accuracy of the SVM was extracted using the *performance* function of the *mlr* package [35]. The accuracy was then compared in a simple linear model (function *lm*). This was performed for each call type and propagation distance separately.

### Information loss with signal attenuation

To investigate the possible loss of information content of the signal due to atmospheric attenuation and SPL loss, we used Beecher’s information statistic, *H*_*s*_ [36], which informs about the information capacity of a signal. To calculate *H*_*s*_, we used all PC scores into the *H*_*s*_ calculation (function *calcHS*, *IDmeasurer* package [37]). This was performed on subsets of calls propagated at different distances (1–1000 m, 10 calculations per call type in total).

## Results

### Apparent sound pressure level

The apparent sound pressure levels, expressed as the mean peak ASPL and mean ASPL RMS, were slightly higher for the *classic* than *single* calls (Table 2). However, the maximum peak ASPL and mean ASPL RMS were similar for the two call types (Table 2).

**Table 2:** Maximum and mean SL values of the call types. All SL values are given in dB re 20 μPa at 1 m.

measure	*classic*	*single*	*p-value*
Mean ASPL peak	60 (SD±4)	54 (SD±7)	<0.001
Max ASPL peak	63	63	-
Mean RMS	45 (SD±4)	42 (SD±7)	<0.01
Max RMS	52	51	-

### Classification to individual

**Fig 5:**
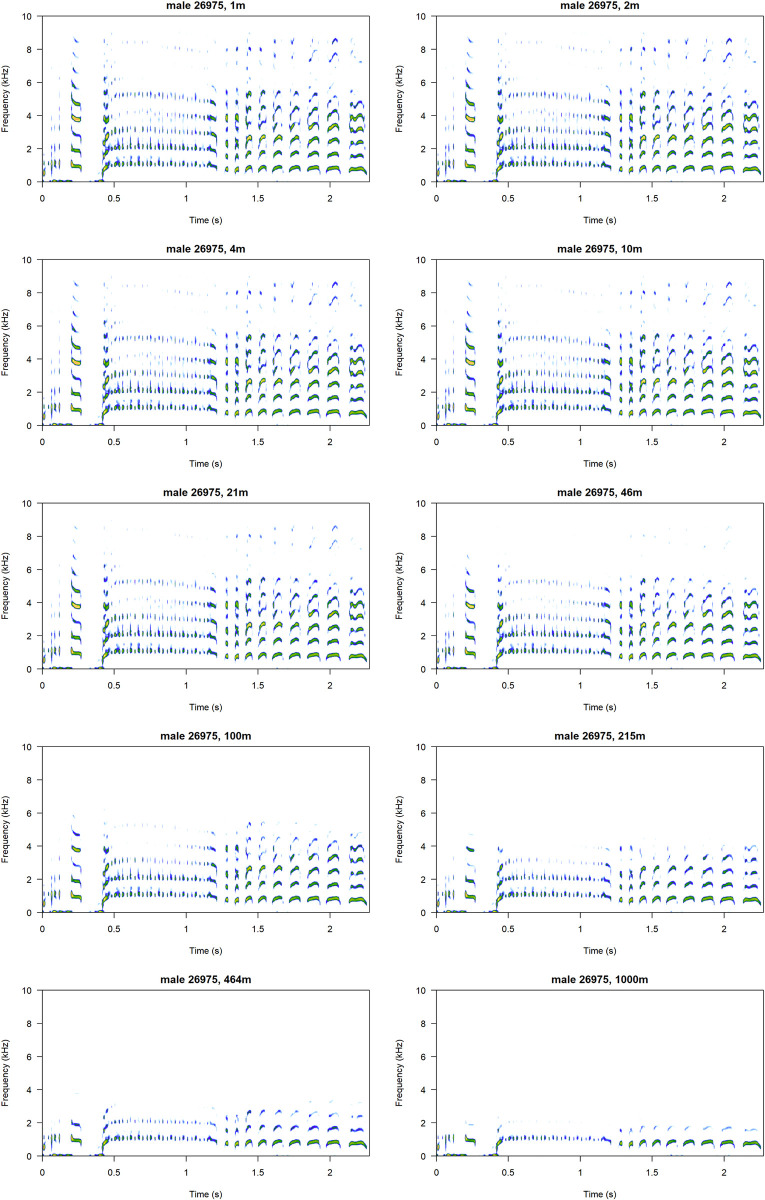
A sample *classic* call attenuated to mimic signal structure at 10 exponential distances in a range of 1–1000 m. Notice that the signal remains very stable, and harmonics are only lost at extreme distances. Note that to improve readability, the spectrograms are not plotted on the same scale, and the colours should not be interpreted as comparable between the panels. Spectrograms plotted using the *seewave* package [12].

**Fig 6:**
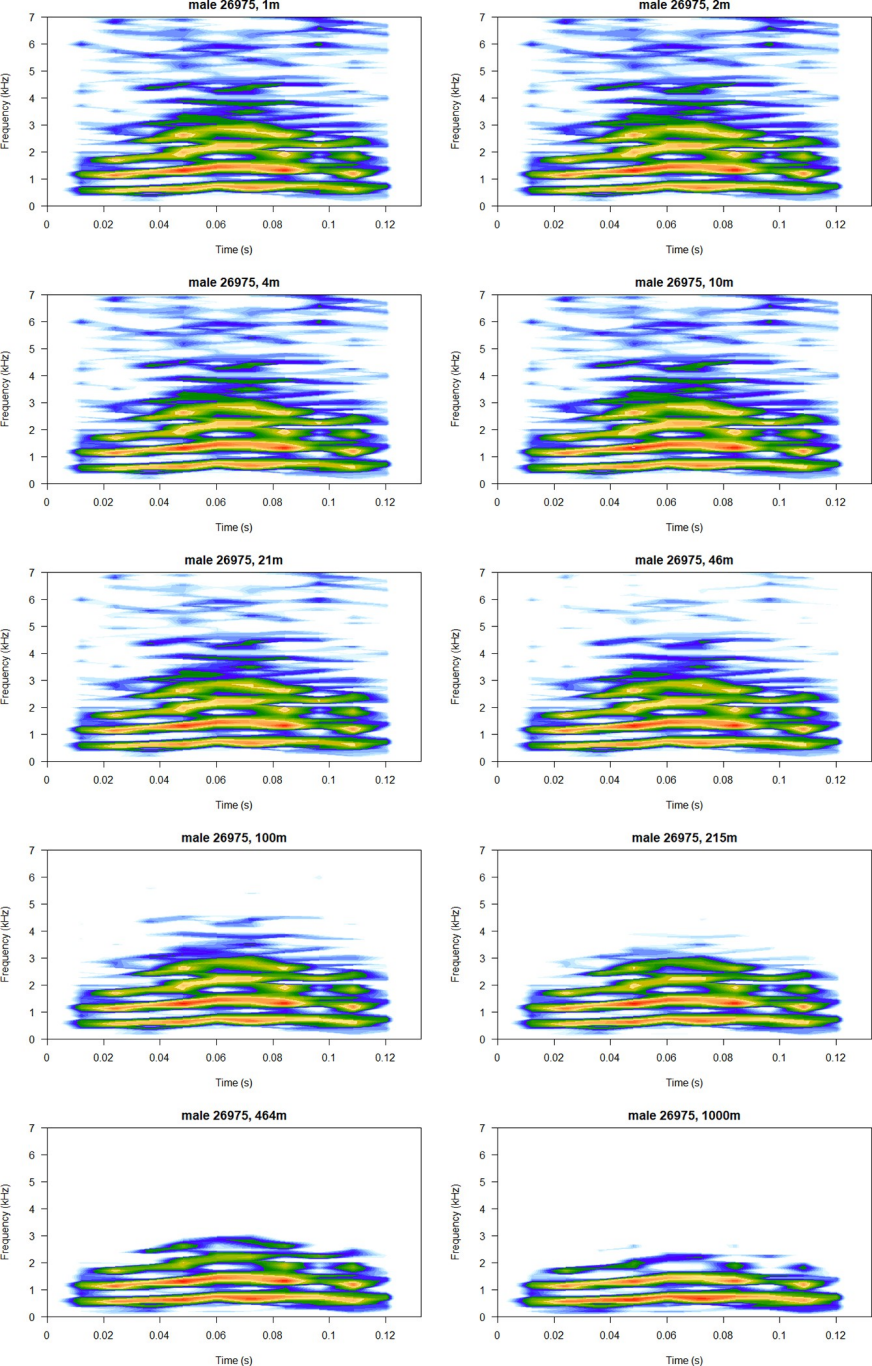
A sample *single* call attenuated to mimic signal structure at at 10 exponential distances in a range of 1–1000 m. Notice that the signal remains very stable, and harmonics are only lost at extreme distances. Note that to improve readability, the spectrograms are not plotted on the same scale, and the colours should not be interpreted as comparable between the panels. Spectrograms plotted using the *seewave* package [12].

**Fig 7:**
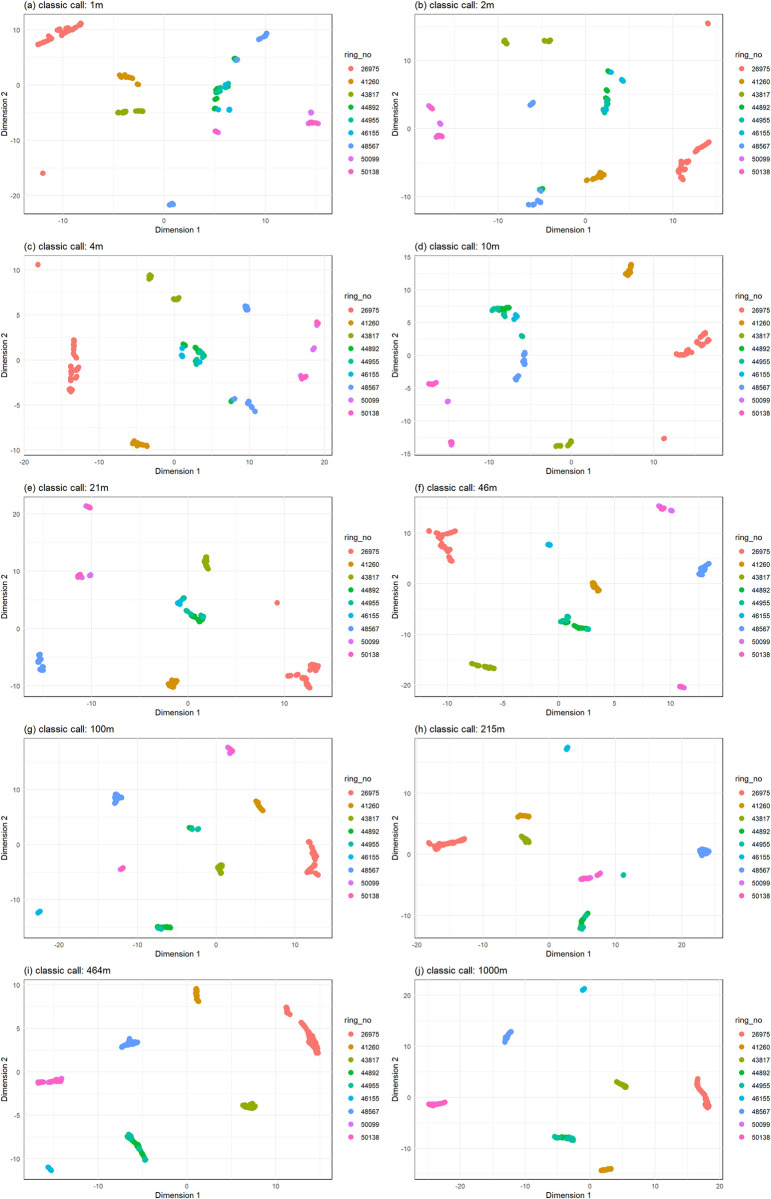
S-UMAP classification of the *classic* call to individual remains efficient in spite of signal filtering and attenuation.

**Fig 8:**
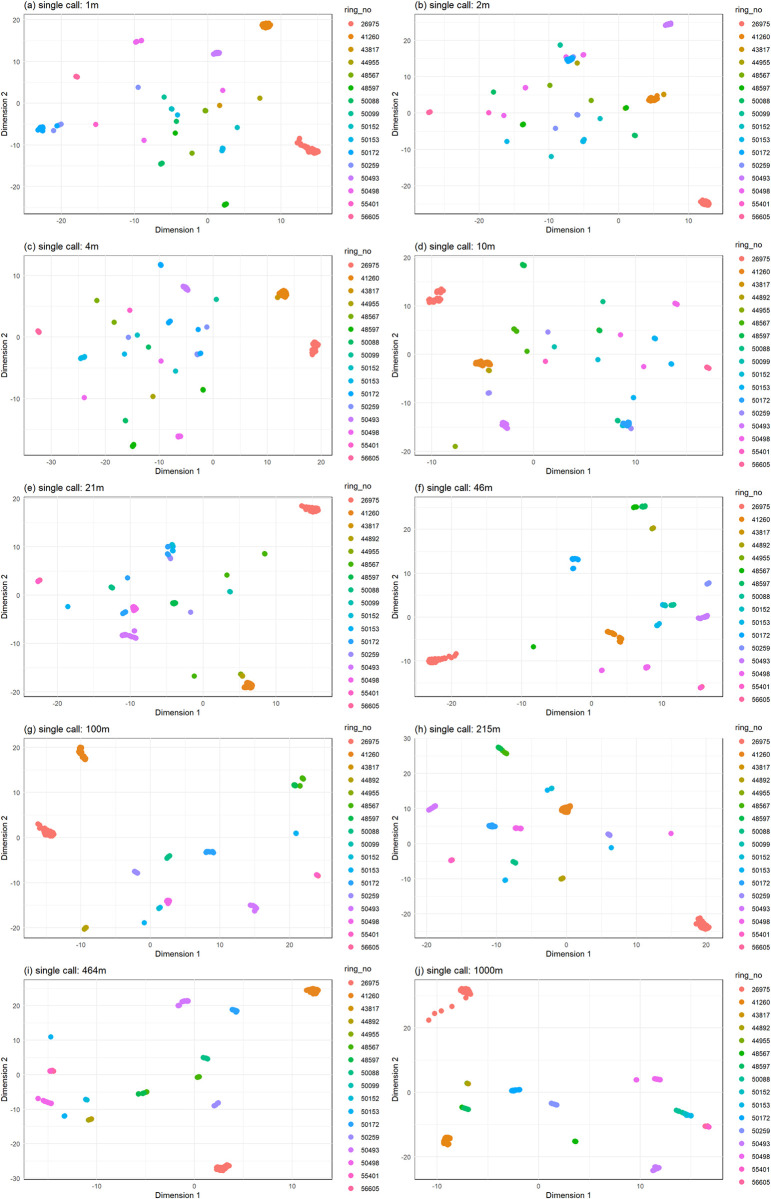
S-UMAP classification of the *single* call to individual remains efficient despite signal filtering and attenuation.

Call structure remained stable independently of signal filtering and attenuation (Figs 5 and 6), and calls could be classified to the correct individual above chance levels (Tables 3 and 4). Clustering accuracy did not decrease with signal attenuation (Figs 7 and 8, and Table 5).

**Table 3:** Results of the permuted discriminant function analysis for *classic* calls attenuated to mimic signal structure at distances from 1 to 1000 m (552 calls of 11 individuals per distance), as well as for all distances pooled together (5520 calls of 11 individuals), using the principal components of eigenvalues > 1. Calls could be reliably classified to individuals above chance levels independently of the attenuation.

Result							
Distance (m)	Correctly classified (%)	Chance level (%)	*p* value for classified	Correctly cross-classified (%)	Chance level for cross-classified (%)	Relative cross-classification level	*P* value for cross-classified
1	49.14	27.83	**0.001**	44.02	9.00	4.89	**0.001**
2	47.56	27.39	**0.001**	44.17	9.06	4.88	**0.001**
4	47.28	27.49	**0.001**	42.74	9.03	4.73	**0.001**
10	47.36	27.70	**0.001**	42.98	9.18	4.68	**0.001**
21	44.96	27.80	**0.001**	42.17	9.26	4.55	**0.001**
46	44.99	28.06	**0.003**	39.45	9.00	4.38	**0.001**
100	45.64	28.06	**0.005**	39.53	9.19	4.30	**0.001**
215	44.61	28.09	**0.002**	40.10	9.17	4.37	**0.001**
464	44.76	27.47	**0.001**	36.20	9.06	4.00	**0.001**
1000	43.79	26.67	**0.001**	31.49	9.09	3.46	**0.001**
pooled	37.79	13.25	**0.001**	47.10	9.05	5.31	**0.001**

**Table 4:** Results of the permuted discriminant function analysis for *single* calls attenuated to mimic signal structure at distances from 1 to 1000 m (264 calls of six individuals per distance), as well as for all distances pooled together (2640 calls of six individuals), using the principal components of eigenvalues > 1. Calls could be reliably classified to individuals above chance level independently of the attenuation.

Result							
Distance (m)	Correctly classified (%)	Chance level (%)	*p* value for classified	Correctly cross-classified (%)	Chance level for cross-classified (%)	Relative cross-classification level	*P* value for cross-classified
1	81.17	40.59	**0.001**	64.84	25.10	2.58	**0.001**
2	76.95	40.75	**0.001**	57.53	23.34	2.47	**0.001**
4	74.43	40.15	**0.001**	57.92	25.40	2.28	**0.001**
10	71.13	40.06	**0.001**	55.26	25.45	2.17	**0.001**
21	75.87	41.12	**0.001**	58.83	25.05	2.35	**0.001**
46	69.27	41.46	**0.001**	53.14	25.69	2.07	**0.001**
100	59.33	41.79	**0.002**	51.49	24.32	2.11	**0.001**
215	75.23	41.77	**0.001**	63.62	21.00	3.03	**0.001**
464	73.43	42.22	**0.002**	62.17	25.98	2.39	**0.001**
1000	70.30	42.11	**0.001**	60.96	25.62	2.38	**0.001**
pooled	54.63	22.40	**0.001**	51.24	19.02	2.69	**0.001**

**Table 5:** Accuracy of classification to individuals using SVM based on S-UMAP reduced data.

Distance	Accuracy [%]	
	*classic* call	*single* call
1	58	73
2	62	72
4	61	85
10	61	69
21	59	74
46	66	78
100	56	83
215	62	77
464	57	89
1000	65	72
p-value	0.4	0.9

### Information loss with signal attenuation

The information capacity of the *classic* call did not decrease as the signal was attenuated and its higher frequency components filtered out, theoretically allowing for a distinction of essentially constant number of individuals as at the source (Table 6). By contrast, the *short* call seemed to be particularly individually specific at a very short range (corresponding to attenuation at 1 m), and maintained roughly 50% of its original information content over further attenuation.

**Table 6:** Beecher’s statistic’s values in the propagated signals. Column *meaning* specifies how many individuals can be theoretically distinguished based on the signal alone.

	*classic call*			*single call*		
Distance [m]	*H*_*s*_ significant	*H*_*s*_ all	meaning	*H*_*s*_ significant	*H*_*s*_ all	meaning
1	2.8	2.8	7	3.5	3.6	12
2	2.8	2.8	6	2.3	2.4	5
4	2.7	2.7	6	2.2	2.2	4
10	2.7	2.7	6	1.9	2.0	3
21	2.8	2.8	7	2.4	2.4	5
46	2.9	2.9	7	3.1	3.1	8
100	2.8	2.7	6	2.5	2.6	5
215	2.8	2.8	7	3.2	3.2	9
464	2.5	2.5	5	2.7	2.7	6
1000	2.8	2.8	6	2.6	2.6	5

## Discussion

We showed that, while the little auk social call is not a particularly loud signal (maximum 63 dBpeak re 20 μPa at 1 m for both call types produced inside the nesting crevice; compared to the loudest species reaching 140–150 dBpeak re 20 μPa at 1 m in air [38]. But see Discussion below on the likely underestimation of this level), it is structured in a way that allows carrying individual information over potentially large distances in the right conditions. Calls could be classified to callers with very similar reliability independently of the higher frequency filtration and attenuation, and well over the likely active space of the signal.

The *classic* call is the longest and most complex of the little auk repertoire [7]. Conspicuous signals are generally thought to have evolved for two main reasons: signalling quality and signal efficacy [39]. The *classic* call certainly matches the latter description, maintaining its carrying capacity over distance. Similarly to other seabird vocalisations [40–43], little auk calls are reliable ‘self-reporting signals’ [44], i.e. they provide information about the signaller. They carry cues to identity, notably in their fundamental frequency [10], unaffected by atmospheric attenuation over distance. However, little auks’ vocal identity can be somehow diluted when considering some parameters, since nesting partners match certain parameters of their calls, such as formant dispersion [9]. From a propagation perspective, as higher frequency formants are attenuated earlier on (see Fig 5), suggesting that partners’ vocalisations become less similar with the loss of higher frequency components of the signal, this may result in a seemingly increasing individual information content as the *classic* call travels further and further.

Long, complex signals can be used in long-distance communication in both humans [45], and non-human animals [39, 46–47]. One aspect of the *classic* call that we did not investigate here is individuality coding within the temporal patterning of the call’s syllables–which in fact holds some of the parameters with the highest potential for individuality coding [10]. This was omitted due to the very heavy workload required to extract this information from such a large dataset. Nevertheless, the fact that strong individuality was retained even when excluding those parameters supports the notion that this call type is “designed” to facilitate efficient communication of identity. Adding the temporal information would very likely further increase the information content measured here, and improve clustering efficiency.

On the other hand, brevity often characterises short-distance communication [46]. The *single* call is a very short, simple signal. While the classification efficiency of this call was essentially similar as the higher frequency components were filtered out, its information content dropped by roughly a half within the first two modelled meters. This may suggest the primary role of this call type may lie more within short-range communication, i.e. to encode private information [47].

Of course, retaining information over distance does not automatically translate into eliciting behavioural reactions to it. For instance, the corncrake *Crex crex*, whose calls carry cues to individuality over long distances [48], but only result in response at behaviourally relevant distances [49], possibly due to the species’ territoriality. However, the little auk’s Umwelt is very different of this of a corncrake, and such efficient long-distance communication could prove particularly useful. For instance, vocalisations could facilitate important aspects of a little auk’s life that might require individual recognition at long-distances, such as communication at foraging grounds, locating one’s neighbours or partner after migration, or even facilitating migratory behaviours. Dedicated studies are necessary to understand whether and how sound might play a role in these behaviours, and how do little auk signals actually propagate over distance.

Long distance communication in the atmosphere is more likely to occur in environments with less physical constraints for sound transmission. For example, open habitats, such as the Arctic tundra or the sea degrade acoustic signals less than closed habitats [23]. However, acoustic communication in the atmosphere is also constrained by a number of factors contributing to signal attenuation, such as air humidity, temperature, and pressure [50]. In response to this, animal signals can evolve to match the optimal frequency ranges for sound communication within their environments. The acoustic adaptation hypothesis (i.e. the notion that the vocal signal of a species will follow their habitat structure, e.g. open/closed) finds only some evidence and only in certain groups [51–52], and a better match between signal properties and the environment can possibly be found at more local scales (as is e.g. the hooded crow, *Corvus cornix [53]*). While the fact that the Arctic tundra, as an open, humid habitat provides excellent conditions for sound propagation is not surprising, the reliability of information transmission found here is noteworthy. Note however, that this should be severely impacted by wind and other environmental noise (discussed below).

So how far away from each other can two little auks be and still recognise the other’s voice, or react to it? This remains unknown, as here, we did not approach any cognitive tests and could only show that the signals themselves can be reliably classified to a sender independently of high frequency filtering, and way over the likely effective distance. This should be considered in the frame of information *content* and *transfer*, and not *meaning* (Weaver’s Levels A and B of communication problems [54]). That is, we cannot and do not intend to suggest to what level do little auks actually decode those transmitted signals and attribute them to individuals they know and recognise. Playback experiments in controlled conditions would be the only way to understand whether and how far away do little auks actually respond to such signals.

While we are not sure yet about the active space of little auk calls, or the distances at which these animals communicate, the fact that signal filtering and attenuation simulated here did not impact the classification to individuals is very interesting. For the birds, this means that the identity information encoded in such signals is structured in a way that makes it not severely impacted by atmospheric attenuation alone. For human observers, such as people employing acoustic capture-recapture methods or studying vocal individuality in the wild, this may be very useful, meaning that recordings obtained at a certain distance might still carry the information needed. In both cases, of course, this will be further impacted by noise.

### Caveats and issues

This study, of course, comes with a number of limitations. While we are confident that the propagation model employing spherical spreading is appropriate for the studied vocalisation (uttered at great heights in an open habitat), it is necessarily simplified and does not correct for subtle changes to air layer densities, wind speed, or topography (see Guibard *et al. [21]*for a brilliant model of ground surface communication in mountain habitats).

This study is unable to tackle signal transmission, due to its idealised conditions, free of environmental noise and wind that surely interfere with the signal in real life: from other birds calling to glaciers calving, there are plenty of other sounds masking the little auk signals in their natural environment. Unfortunately, we were unable to perform propagation experiments due to the great heights and distances involved, and we acknowledge the importance of the local excess attenuation that was hence unaccounted for (see e.g. [53] and [21] for theoretical propagation models confirmed experimentally). While we have attempted to overlay the modelled calls with wind noise, this proved unfeasible at the moment: we suggest that an effort should be taken in the future to either obtain usable, calibrated wind recordings or model the wind noise at different wind speeds and improve the current model. Another question stemming from this is whether and how do little auks adjust their vocal activity to the noise levels and meteorological conditions–this remains to be studied and would require dedicated long-term behavioural observations. Nevertheless, taking into account that the purpose of this study was to investigate the information loss related to frequency filtering as the signals travel through the environment–and not how the animals use or perceive them–we believe that this framework still provides useful insights into the acoustic world of this little understood seabird.

It is also likely that this study underestimates the sound pressure levels of the calls: to be sure about the identity of the caller, we could only use calls produced within the nest. However, calls uttered in open spaces are likely to have a much higher amplitude than those produced in the nest, simply because they may be intended as long-distance signals [47]] or because they need to compensate for the increased noise outside (i.e., the Lombard effect, see e.g. [55]). Little auks’ in-flight calls from the study colony are in fact easily picked up by a human ear at roughly 1 km distance (i.e., the distance between the study colony and the Polish Polar Station in Hornsund). Therefore, our study underestimated the real-life sound pressure levels, and therefore the active space, of these vocalisations when they are produced in flight. While this is unfortunate, we feel more confident reporting under- than over-estimated values. Obtaining direct measurements of little auks vocalising in flight would help us estimate the true SPL of these calls, necessary for playback experiments and/or full propagation models in the future.

Perhaps the biggest issue encountered here is that the recording distance (10 cm) falls within the near field of the lower frequency components of the calls–that is, the distance at which the soundwave is not yet fully developed, and might therefore behave differently [56]. Again, this is because recording the birds inside the nest was the only feasible way of obtaining repeated recordings of known individuals in the field. While the near field should not be an issue for the higher frequency components of the little auk calls, we acknowledge that the recorded properties of the lower components might not fully reflect the actual sound properties at larger distances. This small distance to the microphone has also resulted in some (but not all) of the recorded vocalisations being clipped (see Figs 1 and 2), which may additionally interfere with the results.

### Conclusions

We found that the carrying capacity of the little auk social call does not decrease with high-frequency filtering due to atmospheric attenuation over and beyond the likely behaviourally useful range. While these results do not indicate how the signal propagates or whether this information is actually perceived by the animals, this study suggests that the individual identity in little auk calls is coded in a way that theoretically allows for long-distance communication, and can potentially facilitate important social interactions.

## Supporting information

S1 Table Kaiser-Meyer-Oklin factor adequacy: the overall KMO value for the dataset is middling for both call types, and data suitable for factor analysis. (DOCX)

S2 Table Principal Components Analysis: eigenvalues and proportion of variance. (DOCX)

S3 Table Principal Components Analysis: contributions of raw acoustic parameters to the first five principal components of both call types. (DOCX)
