# Supplementary material for: Long distance calls: Negligible information loss of little auk social vocalisations due to high frequency propagation losses
Source: PLoS Comput Biol. 2024 Dec 2;20(12):e1011961. doi: 10.1371/journal.pcbi.1011961 (PMC11981542; doi:10.1371/journal.pcbi.1011961)
Supplement: S1 Table — (DOCX) [file pcbi.1011961.s001.docx]

**Supplementary Materials**

**Supplementary Table 1.** Kaiser-Meyer-Oklin factor adequacy: the overall KMO value for the dataset is middling for both call types, and data suitable for factor analysis.

| **Raw variable** | *classic call* | *single call* |
| --- | --- | --- |
| Duration | 0.59 | 0.54 |
| AM Env Dep mean | 0.74 | 0.51 |
| AM Env Freq mean | 0.66 | 0.50 |
| AM Ms Freq mean | 0.71 | 0.76 |
| Ampl mean | 0.91 | 0.71 |
| CPP mean | 0.83 | 0.64 |
| Dom mean | 0.53 | 0.63 |
| FM Dep mean | 0.73 | 0.75 |
| Peak Freq mean | 0.83 | 0.91 |
| Pitch mean | 0.55 | 0.66 |
| Q25% | 0.81 | 0.79 |
| Q50% | 0.78 | 0.84 |
| Q75% | 0.76 | 0.72 |
| Spec Centroid mean | 0.73 | 0.71 |
| Spec Slope mean | 0.66 | 0.90 |
| **Overall KMO** | **0.75** | **0.74** |
