# Supplementary material for: Long distance calls: Negligible information loss of little auk social vocalisations due to high frequency propagation losses
Source: PLoS Comput Biol. 2024 Dec 2;20(12):e1011961. doi: 10.1371/journal.pcbi.1011961 (PMC11981542; doi:10.1371/journal.pcbi.1011961)
Supplement: S2 Table — (DOCX) [file pcbi.1011961.s002.docx]

**Supplementary Materials**

**Supplementary Table 2.** Principal Components Analysis: eigenvalues and proportion of variance.

|  | ***classic call*** | | | ***single call*** | | |
| --- | --- | --- | --- | --- | --- | --- |
|  | **Eigenvalue** | **Proportion of variance** | **Cumulative proportion** | **Eigenvalue** | **Proportion of variance** | **Cumulative proportion** |
| **PC1** | 2.20 | 0.32 | 0.32 | 2.31 | 0.36 | 0.36 |
| **PC2** | 1.55 | 0.16 | 0.48 | 1.67 | 0.19 | 0.54 |
| **PC3** | 1.30 | 0.11 | 0.60 | 1.17 | 0.09 | 0.63 |
| **PC4** | 1.09 | 0.08 | 0.68 | 1.09 | 0.08 | 0.71 |
| **PC5** | 1.01 | 0.07 | 0.74 | 1.01 | 0.07 | 0.78 |
| **PC6** | 0.98 | 0.06 | 0.81 | 0.95 | 0.06 | 0.84 |
| **PC7** | 0.88 | 0.05 | 0.86 | 0.87 | 0.05 | 0.89 |
| **PC8** | 0.74 | 0.04 | 0.90 | 0.76 | 0.04 | 0.93 |
| **PC9** | 0.70 | 0.03 | 0.93 | 0.58 | 0.02 | 0.95 |
| **PC10** | 0.60 | 0.02 | 0.95 | 0.48 | 0.02 | 0.97 |
| **PC11** | 0.45 | 0.02 | 0.97 | 0.40 | 0.01 | 0.98 |
| **PC12** | 0.44 | 0.01 | 0.98 | 0.37 | 0.01 | 0.99 |
| **PC13** | 0.43 | 0.01 | 0.99 | 0.33 | 0.01 | 1.00 |
| **PC14** | 0.31 | 0.01 | 1.00 | 0.27 | 0.01 | 1.00 |
| **PC15** | 0.12 | 0.00 | 1.00 | 0.10 | 0.01 | 1.00 |
