# Supplementary material for: Long distance calls: Negligible information loss of little auk social vocalisations due to high frequency propagation losses
Source: PLoS Comput Biol. 2024 Dec 2;20(12):e1011961. doi: 10.1371/journal.pcbi.1011961 (PMC11981542; doi:10.1371/journal.pcbi.1011961)
Supplement: S3 Table — (DOCX) [file pcbi.1011961.s003.docx]

**Supplementary Table 3.** Principal Components Analysis: contributions of raw acoustic parameters to the first five principal components of both call types

|  | ***classic call*** | | | | ***single call*** | | | |
| --- | --- | --- | --- | --- | --- | --- | --- | --- |
| **Raw variable** | **PC1** | **PC2** | **PC3** | **PC4** | **PC1** | **PC2** | **PC3** | **PC4** |
| Duration | -0.10 | -0.50 | -0.30 | -0.01 | 0.28 | -0.72 | -0.34 | 0.23 |
| AM Env Dep mean | 0.01 | 0.66 | 0.42 | 0.15 | -0.26 | 0.65 | 0.62 | -0.11 |
| AM Env Freq mean | -0.31 | 0.19 | 0.42 | -0.64 | 0.35 | -0.08 | -0.56 | -0.52 |
| AM Ms Freq mean | -0.05 | -0.47 | -0.37 | 0.21 | -0.17 | 0.17 | -0.56 | 0.06 |
| Ampl mean | -0.04 | -0.06 | 0.00 | 0.27 | 0.11 | 0.47 | -0.33 | 0.48 |
| CPP mean | -0.68 | 0.20 | -0.03 | 0.54 | 0.42 | 0.53 | -0.30 | 0.37 |
| Dom mean | -0.28 | 0.44 | -0.71 | -0.23 | 0.24 | -0.70 | 0.20 | 0.43 |
| FM Dep mean | 0.04 | -0.73 | 0.05 | -0.36 | 0.30 | -0.30 | -0.21 | -0.25 |
| Peak Freq mean | -0.87 | -0.20 | 0.14 | -0.15 | 0.85 | -0.06 | 0.24 | -0.19 |
| Pitch mean | -0.31 | 0.42 | -0.74 | -0.26 | 0.41 | -0.61 | 0.39 | 0.25 |
| Q25% | -0.83 | 0.27 | -0.01 | 0.12 | 0.81 | -0.11 | 0.08 | -0.29 |
| Q50% | -0.78 | 0.26 | 0.11 | -0.19 | 0.93 | 0.02 | 0.17 | -0.15 |
| Q75% | -0.91 | -0.22 | 0.12 | -0.00 | 0.88 | 0.24 | 0.05 | 0.11 |
| Spec Centroid mean | -0.95 | -0.15 | 0.08 | 0.09 | 0.95 | 0.22 | 0.04 | 0.09 |
| Spec Slope mean | -0.58 | -0.51 | -0.03 | 0.07 | 0.79 | 0.47 | -0.10 | 0.15 |
